# Hypertension and atrial fibrillation: Closing a virtuous circle

**DOI:** 10.1371/journal.pmed.1003598

**Published:** 2021-06-01

**Authors:** Ying X. Gue, Gregory Y. H. Lip

**Affiliations:** 1 Liverpool Centre for Cardiovascular Science, University of Liverpool and Liverpool Heart & Chest Hospital, Liverpool, United Kingdom; 2 Aalborg Thrombosis Research Unit, Department of Clinical Medicine, Aalborg University, Aalborg, Denmark

## Abstract

Ying Gue and Gregory Lip discuss the accompanying study by Ana-Catarina Pinho-Gomes and co-workers on blood pressure lowering treatment in patients with atrial fibrillation.

Atrial fibrillation (AF) is the most common sustained cardiac arrhythmia and is associated with an increased risk of major adverse cardiovascular events (MACE) [1]. Patients with AF typically have other concomitant cardiovascular risk factors—hypertension being one of the commonly associated conditions with a prevalence of up to 90% in major clinical trials of AF [2]. Not only is hypertension common in AF, but it is also an independent risk factor for ischaemic and haemorrhagic strokes, thus bearing important implications for patient prognosis [3]. Therefore, optimal management of hypertension in patients with AF is vital to prevent future MACE. In the accompanying individual-participant data (IPD) meta-analysis [4], the authors from the Blood Pressure Lowering Treatment Trialists’ Collaboration (BPLTTC) aimed to investigate the effects of blood pressure (BP) lowering treatment on MACE when comparing patients with and without AF at baseline. They aimed to address 4 main questions: firstly, whether AF at baseline modifies BP treatment effects; secondly, whether associations between intensity of BP reduction and outcomes are similar with or without AF; thirdly, whether treatment effect is dependent on baseline systolic BP; and lastly, whether classes of antihypertensives have different treatment effect in AF.

A total of 22 trials were included with a total of 188,570 participants and 13,266 patients with a history AF at baseline. Baseline characteristics were different between the 2 groups, with AF patients being older (mean age 70 years versus 65 years), had lower baseline BP (mean 143/84 mmHG versus 155/88 mmHg), and were more commonly prescribed diuretics (50.5% versus 23.8%), angiotensin converting enzyme inhibitors (59.6% versus 44%), beta-blockers (51.3% versus 36%), and alpha-blockers (10.7% versus 4.4%) at baseline. This reflects the more commonly associated cardiovascular comorbidities (hypertension, heart failure, and older age) in patients with AF at baseline [3,5,6].

Among the authors’ findings, firstly, the mean difference in Systolic blood pressure (SBP) reduction was 7.2 mmHg in placebo-controlled studies (8 studies), 2.3 mmHg in drug–drug comparisons (12 studies), and 10.9 mmHg in more-versus-less intensive treatment trials (2 studies) with an overall difference of 3.7 mmHg. When comparing differences in SBP reduction, the authors reported no difference between patients with or without AF (3.3 mmHg versus 3.7 mmHg).

Secondly, meta-regression showed that each 5 mmHg reduction in BP equated to a 10% reduction in MACE in patients with and without AF. Thirdly, authors found no evidence of difference in treatment effects at different baseline systolic BP. And lastly, there was no difference between classes of antihypertensives (renin-angiotensin-aldosterone system inhibitor (RAAS-I) and calcium channel blockers (CCB)), although this conclusion was limited by small numbers of participants with AF in these studies.

The authors conclude that due to the higher risk of MACE in AF patients compared to those without AF, the same relative risk reduction with BP control translates to greater absolute risk reduction in AF patients and, therefore, more focus should be placed on addressing the associated cardiovascular risk factor such as hypertension to better improve the outcomes in patients with AF.

We congratulate the authors for performing this highly relevant IPD meta-analysis to highlight the importance of the holistic management of patients with AF and the need for more evidence in this area. This thought process is echoed in the most recent European Society of Cardiology (ESC) guideline on the management of AF with a shift from managing AF, from the CC (Confirm AF and Characterise AF) to ABC (“A” Anticoagulation/Avoid stroke, “B” Better symptom control, and “C” Comorbidities/Cardiovascular risk factor management) approaches of managing AF [7].

The association of BP control and reduction in MACE in patients with AF does not come as a surprise as hypertension has been linked not only with adverse cardiovascular outcomes but also with an increased risk of AF [8]. The importance of BP control has previously been shown in a large meta-analysis of 61 prospective observational studies involving 12.7 million person-years, i.e., that there is a linear relation between BP and vascular (and overall) mortality, starting from values of 115/75 mmHg [9]. BP control reduces mortality from ischaemic vascular events and haemorrhagic complications from anticoagulation treatment in patients with AF [3]. The reduction in mortality was reflected in the present study [4], although there was no differentiation between haemorrhagic and ischaemic stroke in the outcomes.

One limitation of this work is the inclusion of trials involving only patients with AF. AF status being the inclusion or exclusion criteria prior to randomisation could add to the risk of selection bias within the analysis. In addition, the majority of AF participants are from the Atrial Fibrillation Clopidogrel Trial With Irbesartan for Prevention of Vascular Events (ACTIVE-I) trial dataset [10], which can bias the effect seen given the difference in patient characteristic at baseline. Similarly, including trials with only patients without AF may dilute the effects of BP lowering that could be seen in patients with AF. The authors have addressed this by performing sensitivity analyses excluding these studies which have shown comparable results, reassuring us that the impact of selection bias is not significant on the study conclusions.

In the new 2020 ESC guidelines on the “ABC” approach to the management of AF [7], the management of other associated cardiovascular risk factors has become an integral component of optimal management of AF. The shift in the management of AF towards a more holistic approach is one step in the right direction as has been shown by the improvement in outcomes [11,12] and reduction in healthcare-associated costs [13]. Compliance with the “ABC” management approach requires clear, evidence-based guidelines in terms of treatment targets. With regard to hypertension, the currently recommended BP target (≤130/80) is based upon the current ESC hypertension guidelines [14] and observational data showing greatest benefit of BP between 120 and 129 systolic [15–17]. Whether this target is optimal for the reduction of future MACE in patients with AF is unknown.

This IPD meta-analysis by the BPLTTC has shown that the presence of AF does not alter the treatment effects of antihypertensives. BP lowering in patients with and without AF shows a corresponding reduction in MACE to a similar extent. Owing to the higher absolute risk of MACE in patients with AF, BP lowering in these patients would result in greater absolute risk reduction. This should provide sufficient evidence to convince clinicians regarding the benefits of strict BP control in patients with AF, and the consultation for patients with AF should always involve a conversation about managing hypertension, be it lifestyle modification or pharmacological treatment. However, the potential benefits (or harms) of a much lower BP target (below the recommended 130/80 mmHg) and ideal choice or combination of antihypertensives remain unanswered and would require future studies to provide further insight.

## Abbreviations

ACTIVE-I: Atrial Fibrillation Clopidogrel Trial With Irbesartan for Prevention of Vascular Events
AF: atrial fibrillation
BP: blood pressure
BPLTTC: Blood Pressure Lowering Treatment Trialists’ Collaboration
CCB: calcium channel blockers
ESC: European Society of Cardiology
IPD: individual-participant data
MACE: major adverse cardiovascular events
RAAS-I: renin-angiotensin-aldosterone system inhibitor
SBP: Systolic blood pressure
